# Distended Seminal Vesicles Are Involved in Specific Cerebral Sexual Arousal: A Pilot Study Using Functional Brain Imaging in Young Healthy Men

**DOI:** 10.1016/j.euros.2022.05.008

**Published:** 2022-06-14

**Authors:** Christian Weisstanner, Manuela Pastore-Wapp, Martin Schmitt, Pascal Zehnder, Roland Wiest, George N. Thalmann, Frédéric D. Birkhäuser

**Affiliations:** a Support Center for Advanced Imaging (SCAN), Department of Diagnostic and Interventional Neuroradiology, Inselspital, Bern University Hospital, Bern, Switzerland; b Department of Urology, Inselspital, Bern University Hospital, Bern, Switzerland; c Urologie St. Anna, Lucerne, Switzerland

**Keywords:** Arousal, Brain activity, Functional magnetic resonance imaging, Libido, Seminal vesicles, Sexual activity

## Abstract

**Background:**

Whether seminal vesicles play a role in sexual activity in men is unknown. No study so far has compared the neural processing of visual sexual stimuli in men depending on the filling state of the seminal vesicles.

**Objective:**

To evaluate potential specific cortical activation by visual sexual stimuli with distended and empty seminal vesicles.

**Design, setting, and participants:**

A prospective case-control trial was conducted. Six male individuals underwent two visits on 2 consecutive days for hormone analyses; Derogatis Interview for Sexual Functioning (DISF) questionnaire; functional magnetic resonance imaging (fMRI) with passively viewing sexual, neutral, positive, and negative emotional pictures; and structural pelvic MRI. After the first visit, the participants had to empty seminal vesicles by masturbation. During fMRI, every participant viewed alternating blocks of sexual, neutral, positive, and negative emotional pictures.

**Outcome measurements and statistical analysis:**

Comparisons between days 1 and 2 were evaluated using paired *t* tests.

**Results and limitations:**

No significant differences were observed regarding hormone analyses, DISF questionnaire score, and arousal scoring between days 1 and 2. Seminal vesicle volume was significantly lower on day 2 (*p* = 0.003). Significantly higher activation was observed in the right precentral gyrus, middle frontal gyrus, and right superior temporal sulcus when contrasted for sexual over neutral (*p* < 0.05).

**Conclusions:**

In response to pictures with sexual emotional content, significantly higher activation was detected in brain areas involved in motor preparation (arousal) and coding of desirability of visual sexual stimuli in men with distended seminal vesicles than in the same men with emptied seminal vesicles. This suggests that the filling state of the seminal vesicles may influence sexual desire in men.

**Patient summary:**

We compared brain activity of men with filled and emptied seminal vesicles by functional magnetic resonance imaging. We found that men with filled seminal vesicles had higher activation of brain areas involved in arousal and sexual desire.

## 1 Introduction

The Italian anatomist Gabriel Fallopius first described seminal vesicles in 1561. Seminal vesicles contribute a secretion of various substances to the ejaculate, enabling intact function of spermatozoa. Until today, their physiological role besides their role in fertility is still not entirely understood. Recently, a mouse model suggested an impact of distended seminal vesicles on sex drive in male mice. Male mice with occluded, and thus engorged, seminal vesicles showed a significantly higher sex drive than both mice with resected seminal vesicles and sham operated mice [1].

All organs in the lesser pelvis, in particular the rectum, urinary bladder, seminal vesicles, prostate, urethra, and penis, are innervated from the inferior hypogastric plexus. The inferior hypogastric plexus contains nerves from the sacral plexus and the superior hypogastric plexus, which takes its origin in the thoracic and lumbar segments of the spinal cord [2].

Since the late 20th century, functional neuroimaging techniques have increasingly been used to investigate the neuroanatomical correlates of sexual arousal in healthy humans [3], [4], [5]. Using functional magnetic resonance imaging (fMRI), the sites of cortical activation are reported consistently across studies [6].

The aim of the present study was to evaluate whether the filling state of seminal vesicles is an independent factor influencing sexual arousal in healthy men, that is, whether men with filled, and thus engorged, seminal vesicles have a significantly higher sex drive than men with empty seminal vesicles. We hypothesise that the filling state of seminal vesicles is an independent factor for the intensity of sexual desire, influencing sexual arousal in healthy men.

Therefore, we evaluated cortical activation by fMRI of the brain during visualisation of sexual stimuli under standardised conditions: first after sexual abstention, and thus with distended seminal vesicles, and second after masturbation, and thus with emptied seminal vesicles. Corroborating results in men would imply seminal vesicle-sparing surgery, if cancer stage allows, and may lead to an improvement of surgical techniques, namely, of radical prostatovesiculectomy.

## 2 Patients and methods

### 2.1 Participants

Six healthy heterosexual men between 23 and 29 yr of age (mean 25.9 ± 2.0 yr) were included. All the participants were right handed. The study was performed at the Bern University Hospital, Inselspital, in Bern, Switzerland.

### 2.2 Inclusion and exclusion criteria

The inclusion criteria were male sex, age between 20 and 30 yr, and abstention from ejaculation for 3–5 d before the first fMRI examination. The exclusion criteria were erectile dysfunction, depression, status after brain trauma, claustrophobia, severely reduced visibility, and central nervous system medication.

### 2.3 Endpoints

The primary endpoint was the effect of the filling state of the seminal vesicles on the cortical activation and sexual arousal investigated by fMRI. The secondary endpoints comprised changes in sexual arousal score with filled (day 1) and respectively emptied (day 2) seminal vesicles, and changes in the volume of seminal vesicles before (day 1) and after (day 2) masturbation investigated by structural pelvic MRI.

### 2.4 Operation of trial

**Fig. 1:**
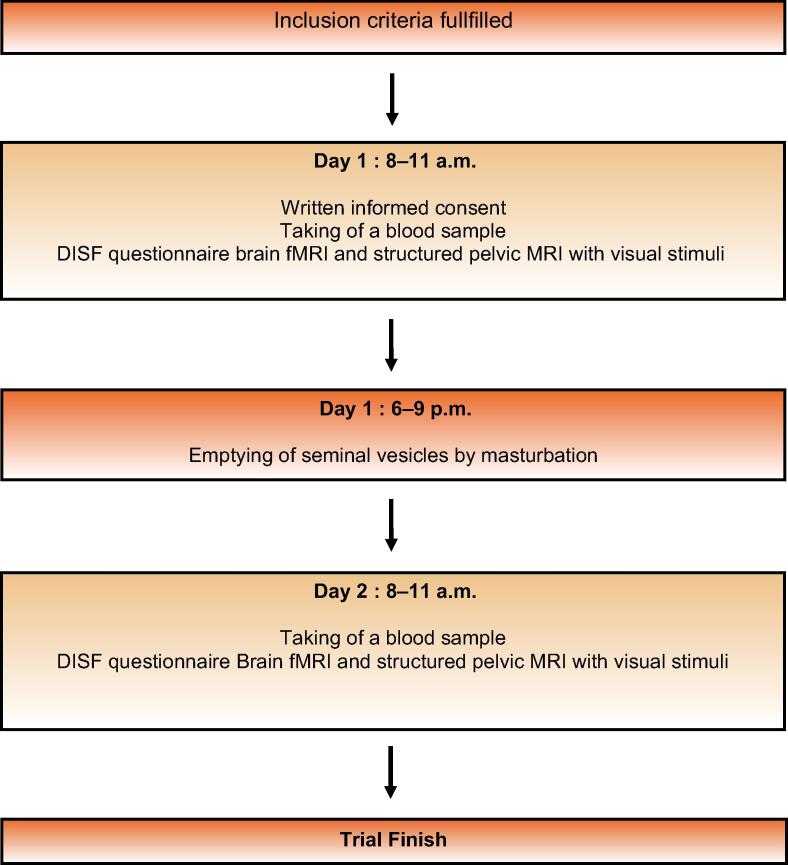
Operation of trial: flowchart. DISF = Derogatis Interview for Sexual Functioning; fMRI = functional magnetic resonance imaging; MRI = magnetic resonance imaging.

All the participants underwent two visits on 2 consecutive days (days 1 and 2) between 8 and 11 a.m. On both visits, first a venous blood sample was taken for the analysis of total and free testosterone, sex hormone binding globulin, prolactin, serotonin, and thyroid stimulating hormone. Thereafter, the Derogatis Interview for Sexual Functioning (DISF) questionnaire [7] was filled out by the participant in a separate room. Then, the fMRI and structural pelvic MRI examinations were performed. All MRI scans were analysed by the same neuroradiologist (C.W.) with over 9 yr of experience in structural and functional imaging. The role of the neuroradiologist is the validation of the generated Montreal Neurological Institute (MNI) coordinates, which are objective measures of the centres of gravity of the activation maps. After acquisition of fMRI, the participants had to rate their arousal during the fMRI in a written form on a score from 1 (none) to 10 (maximum). On day 1 between 6 and 11 p.m., the participants had to empty seminal vesicles by masturbation (Fig. 1).

The study was performed according to the Declaration of Helsinki and was approved by the local ethics committee (Kantonale Ethikkommission Bern, Switzerland). All the participants provided written informed consent before enrolling.

### 2.5 Imaging parameters

Scans were acquired on a whole-body 3 T MRI scanner (Siemens Trio, Erlangen, Germany). A T1-weighted MPRAGE anatomical scan (repetition time [TR] = 2530 ms, echo time [TE] = 2.2 ms, field of view matrix 256 × 256, slice thickness 1 mm), a functional scan using an echo planar imaging–blood oxygen level dependent sequence (TR = 3000 ms, TE = 30 ms, field of view matrix 192 × 192, slice thickness 3 mm) over the brain and a T2-weighted SPACE anatomical scan (TR = 1600 ms, TE = 111 ms, field of view matrix 400 × 400, slice thickness 1 mm) over the pelvis were obtained.

### 2.6 Functional MRI paradigm

All the participants passively viewed alternating blocks of sexual, neutral, positive, and negative emotional pictures during fMRI. Neutral, positive, and negative emotional pictures were taken from the International Affective Picture System (Center for the Study of Emotion & Attention, University of Florida, Gainesville, FL, USA) for sexual stimuli. Pictures were provided by Wehrum et al [8] and were presented in 16 blocks in a pseudorandomised order. One block included eight pictures. Each picture was showed for 3 s, resulting in 6.4 min long fMRI.

### 2.7 Safety parameters

Potential adverse events were clinically assessed continuously during the two study visits and categorised according to the National Cancer Institute Common Terminology Criteria for Adverse Events in grades 1–5 [9].

### 2.8 Statistical analyses

The study was designed as a prospective case-control trial. Statistical analyses regarding arousal rating, blood analyses values, and filling state of the seminal vesicles were conducted using IBM SPSS Statistics 21.0 (SPSS Inc., Chicago, IL, USA). Statistical tests were two sided with a 5% significance level. Comparisons between days 1 and 2 were evaluated using paired *t* tests.

Image data were analysed using SPM12 (Wellcome Trust Centre for Neuroimaging, London, UK) implemented in MATLAB (R2012a). First, on a single-participant level, all functional images were sliced, time corrected, realigned with the first volume of the functional imaging series, and coregistered with the patients’ anatomical images. Second, the functional images were normalised into standard space defined by the MNI template and smoothed with a Gaussian kernel (full width at half maximum of 8 mm). For the calculation of statistical parametric maps, we used the general linear model; the block design was convoluted with haemodynamic response function. Realignment parameters were modelled into the design matrix as regressors, and to remove low-frequency artefacts, a high-pass filter was used at 128 s for model estimation. Statistical maps were calculated for each experiment. The following contrasts of beta-estimates were calculated for each individual: sex-neutral (= contrasts of interest), sex-positive, and sex-negative, as well as positive-neutral, positive-negative, negative-neutral, and negative-positive. For longitudinal differences (day 1 compared with day 2), second-level random-effect analyses for the contrast of interest were conducted using two-sample *t* tests. A one-sample *t* test was applied to explore for group activation. The threshold at voxel level was set at *p* < 0.001, and family-wise error corrected for multiple comparison at cluster level was set at *p* < 0.05. Arousal score, DISF score, and volume of seminal vesicles were used as covariates.

## 3 Results

Filling state of the seminal vesicles measured by structural pelvic MRI was significantly lower on day 2, that is, after emptying by masturbation, than that on day 1 (*p* = 0.003; Table 1).

**Table 1:** Comparison of structural pelvic MRI, hormonal analyses, and questionnaires between days 1 and 2 (paired *t* test)

	Day 1	Day 2	*t* value	*p* value
Seminal vesicles				
Volume (mm^3^)	8328.5 ± 1771.33	4134.5 ± 2388.08	5.34	0.003
Hormone analyses				
Testosterone total (nmol/l)	25.5 ± 13.53	24.27 ± 10.62	0.67	0.53
Testosterone free (pmol/l)	54.7 ± 24.80	53.9 ± 20.74	0.34	0.75
SHBG (nmol/l)	44.0 ± 19.93	42.7 ± 18.09	1.04	0.36
Prolactin (µg/l)	13.4 ± 4.12	14.8 ± 5.07	–1.02	0.36
Serotonin (µg/l) a	<10 ± 1.75	<10	1.00	0.39
TSH (µU/ml)	2.2 ± 1.09	2.4 ± 0.79	–0.79	0.46
Questionnaires				
Arousal b	7.5 ± 1.37	7.33 ± 1.86	0.54	0.61
DISF	59.3 ± 6.47	53.5 ± 13.00	1.74	0.14

DISF = Derogatis Interview for Sexual Functioning (*t* values); MRI = magnetic resonance imaging; SHBG = sex hormone binding globulin; TSH = thyroid stimulating hormone.

Values are mean ± standard deviation; *p* values are two sided.

a Serotonin analysis is missing in two participants on day 2.

b Sexual arousal questionnaire: 1 = no arousal, 10 = high arousal.

Analyses of total testosterone, free testosterone, sex hormone binding globulin, prolactin, serotonin, and thyroid stimulating hormone did not differ between days 1 and 2 (Table 1).

There was no significant difference in the arousal score (*p* = 0.611) and DISF questionnaire score (*p* = 0.14) between days 1 and 2 (Table 1). In addition, the subscores sexual cognition and fantasy score, sexual arousal score, sexual behaviour and experience score, orgasm score, and sexual drive and relationship score revealed no significant differences between days 1 and 2 (for all scores, *p* > 0.34).

**Fig. 2:**
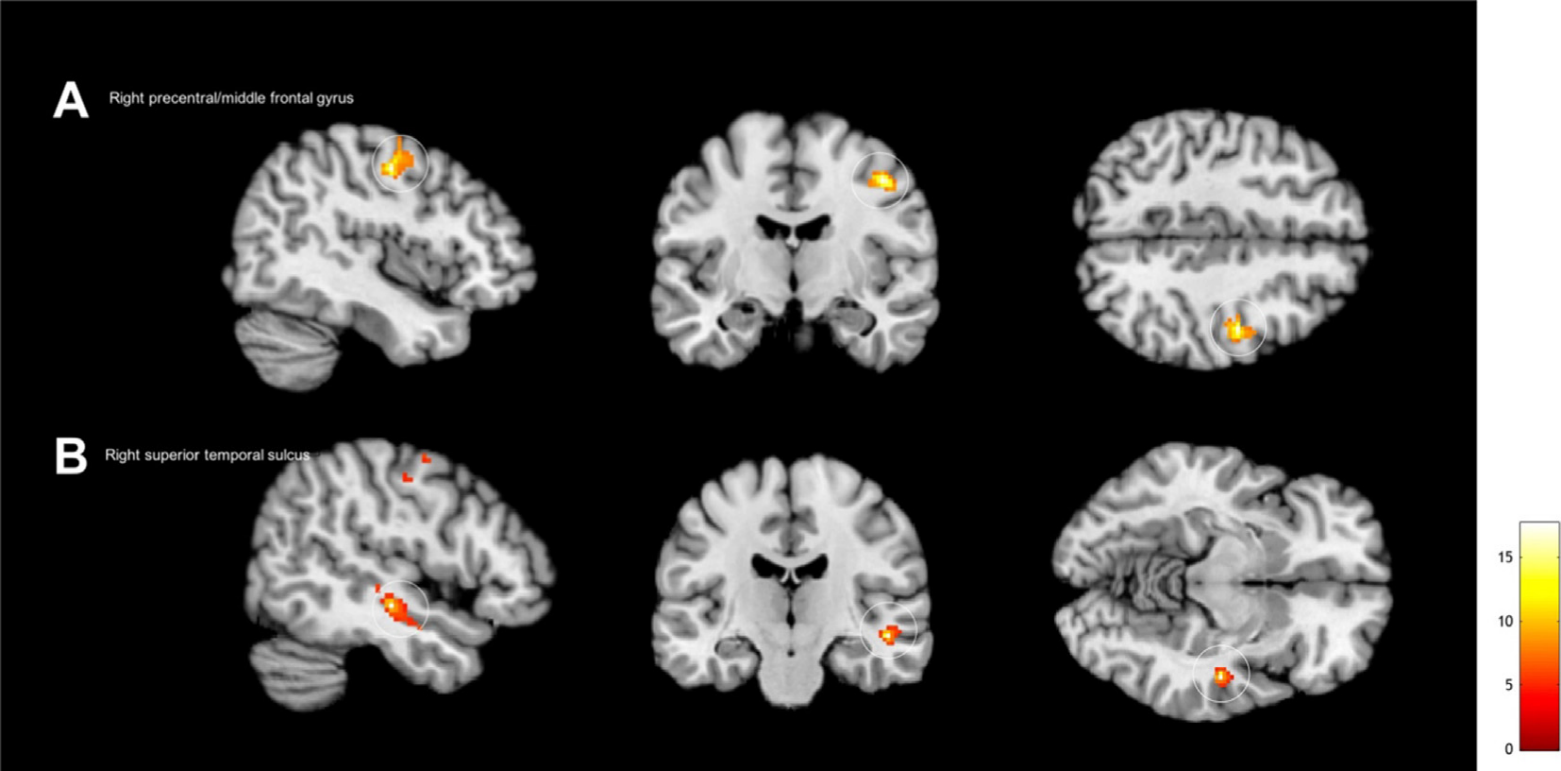
Whole brain analysis revealed significantly (voxel level was set at *p* < 0.001 and family-wise error corrected for multiple comparison [*p* < 0.05] at cluster level) higher activation in the (A) right precentral gyrus and middle frontal gyrus (Brodmann area 6) and (B) right superior temporal sulcus when contrasted for sex over neutral on day 1, when compared with day 2.

In the fMRI experiment, on day 1 when compared with day 2, a whole brain analysis revealed significant higher activation in the right precentral gyrus and middle frontal gyrus (*x* = 50, *y* = –18, *z* = –10, *T* = 17.62; *p* = 0.015; Brodmann area = 6; Fig. 2A) and right superior temporal sulcus (*x* = 44, *y* = –10, *z* = 46, *T* = 10.39; *p* = 0.031; Fig. 2B) when contrasted for sex over neutral (Table 2).

**Table 2:** Regions of significant higher activation when contrasted for sex over neutral on day 1 when compared with day 2

Comparison day 1 versus day 2: sex-neutral								
Macroanatomical region	Contrast	Cluster level		Peak level				
		Size	*P*_FWE_	Coordinates				T
					*x*	*y*	*z*	
*Positive activation*								
Right superior temporal gyrus	Sex-neutral	134	0.015		50	–18	–10	17.62
Right precentral gyrus	Sex-neutral	114	0.031		44	–10	46	10.39

MNI = Montreal Neurological Institute.

Reported coordinates refer to MNI space; *P*_FWE_ = family-wise error corrected for multiple comparison (*p* < 0.05).

In the group analysis (days 1 and 2 taken together), in sex-neutral contrast, significant positive activation was found in the right middle temporal gyrus, left middle occipital gyrus, left fusiform gyrus, right posterior cingulate cortex, and left supramarginal gyrus. Deactivation as a sign of functional inhibition could be found in the right hippocampus, left lingual gyrus, right superior temporal gyrus, right insula, left middle temporal gyrus, and left rolandic operculum (Table 3).

**Table 3:** Regions of significant positive activation and deactivation in group analysis (days 1 and 2 taken together)

Macroanatomical region	Contrast	Cluster size	Peak level MNI			
			*Z*	*x*	*y*	*z*
*Group analysis: sex-neutral*						
Positive activation						
Right middle temporal gyrus	Sex-neutral	2398	6.17	46	–70	0
Left middle occipital gyrus	Sex-neutral	801	5.59	–44	–76	12
Left fusiform gyrus	Sex-neutral	850	5.58	–42	–58	–18
Right posterior cingulate gyrus	Sex-neutral	137	4.69	6	–44	26
Left supramarginal gyrus	Sex-neutral	165	3.80	–54	–30	34
Deactivation						
Right hippocampus	Sex-neutral	366	5.58	32	–40	–4
Left lingual gyrus	Sex-neutral	368	4.68	–28	–48	–2
Right superior temporal gyrus	Sex-neutral	371	4.51	68	–20	8
Right insula	Sex-neutral	359	4.02	34	–16	14
Left middle temporal gyrus	Sex-neutral	286	3.92	–66	–20	2
Left rolandic operculum	Sex-neutral	289	3.87	–46	–20	18
*Group analysis: sex-positive*						
Positive activation						
Left middle occipital gyrus	Sex-positive	2372	6.15	–52	–74	4
Right middle occipital gyrus	Sex-positive	3513	5.46	40	–74	4
Left parietal inferior gyrus	Sex-positive	474	5.07	–34	–50	56
Left superior occipital gyrus	Sex-positive	207	4.63	–20	–84	34
Right parietal superior gyrus	Sex-positive	454	4.62	22	–56	66
Deactivation						
Right parietal inferior gyrus	Sex-positive	387	5.45	52	–48	54
Left parietal inferior gyrus	Sex-positive	180	4.90	–58	–48	46
*Group analysis: sex-negative*						
Positive activation						
Left middle temporal gyrus	Sex-negative	1085	4.77	–40	–66	12
Right anterior cingulate gyrus	Sex-negative	187	4.65	0	28	28
Left anterior cingulate gyrus	Sex-negative	287	4.60	–2	52	10
Left parietal superior gyrus	Sex-negative	215	4.31	–34	–54	58
Right middle occipital gyrus	Sex-negative	1131	4.29	50	–76	0

MNI = Montreal Neurological Institute.

When examining the sex-positive contrast, positive activation was found in the left and right middle occipital gyrus, left parietal inferior gyrus, left superior occipital gyrus, and right parietal superior gyrus. Deactivation occurred in the right and left parietal inferior gyrus (Table 3).

For the sex-negative contrast, positive activation was seen in the left middle temporal gyrus, right and left anterior cingulate cortex, left parietal superior, and left middle occipital gyrus (Table 3). No significant deactivation was found.

## 4 Discussion

The present study provides evidence that the filling state of the seminal vesicles has an influence on functional brain activity in young men. The findings that filled seminal vesicles after sexual abstention increase activity in brain areas involved in motor preparation (arousal) and coding of desirability of visual sexual stimuli suggest that sexual abstention with filled seminal vesicles may increase sexual desire in men. The findings of the present study in men are in line with the conclusions from our earlier mouse model experiment, which revealed that obstructed and thus congested seminal vesicles have a significant effect on sex drive in mice [1].

Several studies examined the neural activity in response to sexually explicit pictures [3], [4], [8], [10], [11], [12], [13], [14], [15], [16], [17], [18], [19], [20], [21]. A quantitative meta-analysis performed by Poeppl et al [6] on 18 MRI and two positron emission tomography studies confirmed the activation of specific brain regions during sexual arousal. The specific brain regions were shown to be involved in modulation of attention and sensory processing, relevance detection and affective evaluation, and inducement of a conscious sexual urge. Our results of the group analysis are consistent with their findings.

To our knowledge, this is the first study that performed a longitudinal analysis of neural activity in response to sexually explicit pictures in relation to the filling state of the seminal vesicles. With respect to the right precentral gyrus and middle frontal gyrus, the activation of these motor and premotor areas is described in some publications covering the topic of functional neuroimaging studies of sexual arousal and may be a sign of desire of performing sexual actions, which may have been revealed by these studies [18].

The right precentral gyrus, middle frontal gyrus, and right superior temporal sulcus are areas not typically associated with arousal. The superior temporal sulcus is a densely connected region and is involved in social cognition, attention, integration of body-related information, and self-processing [22]. Ortigue and Bianchi-Demicheli [23] investigated the responses of nine men and four women to photographic visual stimulus. This study suggested that the superior temporal sulcus plays a crucial role for coding of desirability of visual sexual human stimulus within the first 200 ms after stimulus onset. Wehrum et al [24] could verify the stability of neural responses to visual sexual stimuli over a time span 1–1.5 yr, which could explain why we did not find significant differences between days 1 and 2 in activation in brain areas typically associated with arousal elicited by visual sexual stimuli.

In the present study, structural pelvic MRI showed a significant lower volume of the seminal vesicles on day 2 than on day 1, that is, after emptying seminal vesicles by masturbation. Hormone analyses showed comparable values on both days. Thus, there does not seem to be a hormonal confounder according to the five analysed hormones known to influence sexual desire. Furthermore, scientifically no correlation between sexual abstention with filled seminal vesicles and these hormone levels is known. The DISF questionnaire score before and after emptying seminal vesicles revealed no differences in relation to the two consecutive MRI examinations. Similarly, the arousal during the fMRI examination did not reveal a significant difference between the two MRI examinations. However, these findings assume a comparable objective individual hormonal and psychosexual status before and after the emptying of seminal vesicles. The DISF-SR measures the quality of an individual's sexual functioning within the last month with a high test-retest reliability (0.86) [7]. Further studies should include continuous measures for the degree of sexual arousal, allowing for the calculation of an index of the temporal relationship [25].

The time interval between emptying of seminal vesicles by masturbation and the second structural pelvic MRI and fMRI of the brain was set at 14–16 h. A shorter interval between masturbation and the second MRI examination might confound the results by the physiological postorgasmic refractory period in which male sexual excitement is significantly reduced or impossible. A refractory period can last from a few minutes to several days. The precise length of a refractory period is unknown, and depends mainly on the individual age and frequency of sexual activity [25]. Moreover, it is not known whether such a refractory phase may influence brain activity in a certain manner also after subjective resumption. A longer interval between masturbation and the second MRI examination would potentially allow the seminal vesicles to refill and thus reduce the difference in their filling state. This again would obviously reduce or nullify the hypothesised impact of filled seminal vesicles on the brain. However, structural pelvic MRI documented a significantly reduced volume of the seminal vesicles between the first and second MRI examinations.

A potential limitation of the present study is the small number of participants. Study design and recruitment for this pilot trial turned out to reveal the intrinsic risk of limited recruitment capabilities due to the topic itself. Another potential limitation is the set time interval between masturbation and second MRI. As stated above, the length of this interval may influence the results, and thus must be considered in the present study and in future studies as a potential limitation.

Whether the filling state of the seminal vesicles or the seminal vesicles as anatomic structures themselves have an impact on sexual activity in men is not known. To answer this question, further clinical studies are necessary. However, the present findings might be an incentive to further explore the correlation between seminal vesicles and sex drive.

## 5 Conclusions

In response to pictures with sexual emotional content, significantly higher activation was detected in brain areas involved in motor preparation (arousal) and coding of desirability of visual sexual stimulus in young men under ejaculation abstention, and thus with distended seminal vesicles, compared with the same men after ejaculation, and thus with emptied seminal vesicles. This suggests that the filling state of the seminal vesicles may have an influence on brain activation and sexual desire in men.

  ***Author contributions*:** Frédéric D. Birkhäuser had full access to all the data in the study and takes responsibility for the integrity of the data and the accuracy of the data analysis.

*Study concept and design*: Birkhäuser, Weisstanner, Thalmann.

*Acquisition of data*: Birkhäuser, Weisstanner, Pastore-Wapp, Schmitt, Wiest.

*Analysis and interpretation of data*: Birkhäuser, Weisstanner, Pastore-Wapp, Schmitt, Zehnder, Wiest, Thalmann.

*Drafting of the manuscript*: Birkhäuser, Weisstanner, Pastore-Wapp, Zehnder.

*Critical revision of the manuscript for important intellectual content*: Birkhäuser, Weisstanner, Zehnder, Wiest, Thalmann.

*Statistical analysis*: Weisstanner, Pastore-Wapp, Birkhäuser.

*Obtaining funding*: Birkhäuser, Thalmann.

*Administrative, technical, or material support*: Schmitt, Zehnder.

*Supervision*: Birkhäuser, Thalmann, Wiest.

*Other*: None.

  ***Financial disclosures:*** Frédéric D. Birkhäuser certifies that all conflicts of interest, including specific financial interests and relationships and affiliations relevant to the subject matter or materials discussed in the manuscript (eg, employment/affiliation, grants or funding, consultancies, honoraria, stock ownership or options, expert testimony, royalties, or patents filed, received, or pending), are the following: None.

  ***Funding/Support and role of the sponsor*:** We thank the Max und Hedwig Niedermaier Foundation, Zurich, Switzerland, for financial support. The sponsor played a role in the design and conduct of the study.
